# INTAKE OF SUGAR-SWEETENED BEVERAGES, MILK AND ITS ASSOCIATION WITH BODY MASS INDEX IN ADOLESCENCE: A SYSTEMATIC REVIEW

**DOI:** 10.1590/1984-0462/;2018;36;1;00010

**Published:** 2018

**Authors:** Ana Carolina Corrêa Café, Carlos Alexandre de Oliveira Lopes, Rommel Larcher Rachid Novais, Wendell Costa Bila, Daniely Karoline da Silva, Márcia Christina Caetano Romano, Joel Alves Lamounier

**Affiliations:** a Universidade Federal de São João del-Rei (UFSJ), Divinópolis, MG, Brasil.

**Keywords:** Adolescent, Beverages, Obesity, Body mass index, Adolescente, Bebidas, Obesidade, Índice de massa corporal

## Abstract

Objective: To systematize literature references addressing the association of sugar-sweetened beverage (SSB) and milk intake with body mass index (BMI) in adolescents.

**Data source:**

A search was carried out in PubMed (US National Library of Medicine National Institutes of Health) and BVS (Virtual Library in Health). The descriptors used were: adolescents, young adult, beverages, drinking, obesity, overweight, BMI, and nutritional status. The following filters were applied: age ranging from 10 to 19 years, studies published in Portuguese or English language between 2011-2015.

**Data synthesis:**

Thirty studies were selected (22 cross-sectional studies, 4 cohort studies, 1 randomized clinical trial, 1 case-control study, and 1 quasi-experimental study). There was association between the intake of these beverages and increase in BMI in 55% of all 20 studies that dealt with sugary drinks. When it came to soft drinks, 100% of studies reported association with increase in BMI. As to milk intake, only one article showed association with increased BMI. Three articles reported milk as a protection factor against increase in BMI; three studies found no association between this intake and BMI. Nineteen studies had representative samples and 20 surveys reported random samples. Among papers using questionnaires, 84% had been validated.

**Conclusions:**

There is no consensus in the literature about the association between SSB or milk intake and BMI in adolescents.

## RESUMO

**Objetivo:**

Sistematizar as referências da literatura relacionadas à associação entre consumo de bebidas açucaradas não alcoólicas, leite e índice de massa corporal (IMC) em adolescentes.

**Fontes de dados:**

Realizou-se a pesquisa dos artigos nos portais PubMed (US National Library of Medicine National Institutes of Health) e BVS (Biblioteca Virtual em Saúde). Os descritores foram: adolescente, adulto jovem, bebidas, ingestão de líquidos, obesidade, sobrepeso, IMC e estado nutricional. Utilizaram-se os filtros: idade entre 10 e 19 anos e artigos em português e inglês, publicados entre 2011 e 2015.

**Síntese dos dados:**

Trinta estudos foram selecionados (22 transversais, 4 coortes, 1 ensaio clínico aleatório, 1 caso-controle e 1 quase experimental). Dos 20 estudos que abordaram bebidas açucaradas em geral, 55% encontraram associação entre consumo e aumento do IMC. Em relação aos estudos sobre a ingestão de refrigerantes, todos apresentaram associação entre consumo e aumento do IMC. Dos estudos sobre leite, somente um demonstrou associação entre consumo e aumento do IMC. Três artigos mostraram proteção entre consumo de leite e aumento do IMC, e três trabalhos não encontraram associação de seu consumo com aumento do IMC. Dezenove estudos possuíam amostras representativas e outros 20 declararam ter amostras aleatórias. Dos artigos com questionários, 84% eram validados.

**Conclusões:**

Verifica-se que não há consenso na literatura pesquisada sobre a associação entre consumo de bebidas açucaradas não alcoólicas, leite e IMC de adolescentes.

## INTRODUCTION

Over the last decades, the world - Brazil included - has seen changes in food and nutritional patterns, which has resulted in increased obesity and reduced malnutrition rates. The phenomenon of epidemiological and nutritional transition affects both children and adolescents.^1^ Among factors related, changes in lifestyle and eating habits play an important role. Among other foods and beverages consumed by the population, a greater intake of sugar-sweetened non-alcoholic beverages has been seen. At the same time, the intake of milk and dairy products has decreased, especially among adolescents.^2^ ^,^ ^3^ ^,^ ^4^ ^,^ ^5^

Among environmental causes of obesity in adolescence, changes in their nutritional pattern motivated by political and economic transformations are pointed out and occur in all nations. The strong trend towards consumption of processed and ultra-processed foods and beverages is broad. Special emphasis should be given to the high consumption of sugary drinks. The international literature on the topic includes papers addressing soft drinks, sweetened juices, powdered juices, boxed juices, sports drinks, water, sweetened tea and energy drinks fitted within the category of sugar-sweetened beverage (SSB).^6^ ^,^ ^7^ The excessive intake of this type of beverage is one of the main factors contributing to the obesity epidemic in Brazil and across the world.^2^ ^,^ ^3^ ^,^ ^5^ ^,^ ^8^

In this scenario of changes, the intake of soft drinks in Brazilian households has increased 400% from 1975 to 2003 and 16% from 2003 to 2009.^4^ This considerable increase is alarming, as there is evidence that this type of drink is associated with higher calorie intake and excessive weight gain in adolescents.^8^ As to milk, consumption rates were shown to reduce in 40% between 1975 and 2003 and in 10% between 2003 and 2009.^4^

A likely explanation for sugary drinks to increase body mass index (BMI) is the fact that the intake of liquid carbohydrates causes less satiety than solid carbohydrates, which leads to higher total energy consumed.^9^ On the other hand, several studies in the literature suggest that milk and dairy products in whole versions are protective against obesity, which is explained by the influence of protein, calcium, and fat-soluble vitamins mechanism of action, and by the fact that they induce more satiety.^10^ ^,^ ^11^ ^,^ ^12^ ^,^ ^13^

Adolescents are influenced in school, by friends and the media, which directly interferes in the formation of their eating habits.^14^ Eating inadequacies at this age can determine how puberty will evolve, with growth rate delay and even interruption.^15^ At this stage of life, adolescents start gaining more autonomy, deciding what, when and how to eat. Eating out comes accompanied by modifications. Snacks over main meals, breakfast omission and low consumption of fruits, vegetables, milk, and dairy products can pose consequences such as inadequate calcium, fiber and water intake.^16^

The strength of media and social valorization of ultra-processed foods, nowadays accessible to a large portion of the population, stimulate the consumption of high-calorie products.^17^ Among the wide range of food available, beverage was the topic chosen for our study because it is currently and constantly present in discussions published in the literature and in the media.^18^ ^,^ ^19^ ^,^ ^20^ ^,^ ^21^ Brazilian non-alcoholic beverage companies agreed by consensus not to air advertises about soft drinks and artificial juices for children up to 12 years of age.^19^ In addition, the end of commercialization of these drinks in schools across the country is in discussion.^18^ According to these companies, the end of advertising aimed at this age group is a global trend, inspired by other countries’examples.^19^ In Brazil, draft bills on the subject, such as 4,910/2016 and 1,755/2007, are in the pipeline.^22^ Market research has shown that there will be a drop in consumption of soft drinks in the future, and companies seem to be preparing for it commercially.^18^

Sugary drinks is a theme in ascendancy in foreign research, but is still little discussed in Brazil. Despite the large number of international papers addressing the topic, there is still no consensus on the relationship between intake of sugar-sweetened non-alcoholic drinks or milk and obesity in adolescents. The aim of this study was to systematize the literature references studying the association between intake of sugar-sweetened non-alcoholic drinks or milk and BMI in adolescents. This research may support clinical practice of pediatric health professionals and collaborate with further studies, the differential being the systematic review that evaluates whether a questionnaire on the consumption of certain beverages has been validated, and the analysis of evidence level according to study design, sample randomness and representativeness in the papers selected.

## METHOD

This is a systematic literature review. The search of relevant articles was carried out in July 2015, on the following databases Lilacs (Latin American and Caribbean Literature in Health Sciences), MEDLINE (Medical Literature Analysis and Retrieval System Online), and Ibecs (Spanish Bibliographical Index of Cheers). PubMed (US National Library of Medicine National Institutes of Health) and VHL (Virtual Health Library) were also used. Descriptors used were included in the list of Descriptors in Health Sciences (DeCs) and Medical Subject Headings (MeSH). The boolean operators were “or” and “and”. The following combinations were used in Portuguese: (*adolescente* OR *adulto jovem*) AND (*bebidas* OR *ingestão de líquidos*) AND (*obesidade* OR *sobrepeso*) AND (*índice de massa corporal* OR *estado nutricional*); in English, combinations were: (adolescent OR young adult) AND (beverages OR drinking) AND (obesity OR overweight) AND (body mass index OR nutritional status). Later on, filters were applied: age range between 10 and 19 years, as recommended by the World Health Organization (WHO); and articles published in Portuguese and English between January 1, 2011 and July 31, 2015, when the last search was made.

Critically and independently selected publications were evaluated by two authors. Doubts about the selection of articles were evaluated and agreed among researchers until a consensus was reached. In order to classify the level of evidence of articles, the categorization by the Agency for Healthcare Research and Quality (AHRQ) of 2016^23^ was used, according to which level 1 is considered the greatest strength of evidence and includes meta-analyzes of multiple controlled studies. Individual projects with experimental design, such as randomized clinical trials, are considered as level 2. Cohort studies, case-control and quasi-experimental studies, such as non-randomized studies, are classified as level 3. Non-experimental studies such as cross-sectional ones are sorted as level of evidence 4. Case reports are level 5, and opinions of reputable authorities based on clinical competence or opinions of expert committees and interpretations of non-research based information are at level 6.^23^

The search strategy was conducted according to the proposal by PRISMA (Preferred Reporting Items for Systematic Reviews and Meta-Analyzes, 2009) and to PICOS eligibility criteria (participants, intervention, comparison, outcomes - results - and study design).^24^ From the guiding question “Is there an association between consumption of sugar-sweetened non-alcoholic drinks or milk and BMI in adolescents”, PICOS criteria were: participants of both genders and aging 10 to 19 years. The intervention considered was consumption of sugar-sweetened non-alcoholic beverages, such as juice and soda, or milk. As for comparison, the intake or no intake of the beverages in question was observed. For outcomes, it was analyzed whether the consumption of the mentioned beverages had relation with increase in BMI. In cases of association, it was verified whether it was direct or inverse. In direct association, beverage consumption increased BMI. Therefore, in inverse association, consumption of beverages reduced BMI. Therefore, the studies selected were inputted to three tables taking into account their design and level of evidence.^23^

Table 1 covers articles with cross-sectional studies exclusively on sugar-sweetened beverage. Table 2 shows cross-sectional articles dealing with sugar-sweetened beverage and milk consumption. In Table 3 lists all articles with other study designs.

**Table 1:** Cross-sectional studies (n=15) that analyzed the association of sugar-sweetened beverages intake with body mass index in adolescents.

Reference	Sample size and age groups	Food survey and validations	Association between beverage intake and body mass index
Al-Hazzaa et al., 2011^34^	n=2,908 14-19 years	Questionnaire*	Sugar-sweetened beverages: Inverse association
Al-Hazzaa et al., 2012^10^	n=2,906 14-19 years	Questionnaire*	Sugar-sweetened beverages: Inverse association
Danyliw et al., 2012^35^	n=10,038 2-18 years	24-hour record	Soft drink: Direct association
Jia et al., 2012^36^	n=702 11-15 years	24-hour record	Sugar-sweetened beverages: Direct association
Liu et al., 2012^37^	n=2,286 12-19 years	24-hour record	Sugar-sweetened beverages: Direct association
Emandi et al., 2013^38^	n=3,626 7-18 years	Questionnaire**	Sugar-sweetened beverages: Direct association
French et al., 2013^30^	n=1,015 16-65 years	Questionnaire*	Soft drink: Direct association
Sluyter et al., 2013^39^	n=5,714 12-22 years	Questionnaire**	Soft drink: Direct association
Wate et al., 2013^40^	n=6,871 13-18 years	FFQ*	Sugar-sweetened beverages: Inverse association
Chan et al., 2014^41^	n=2,727 12-16 years	FFQ**	Sugar-sweetened beverages: Direct association
Chan et al., 2014^42^	n=200 12-16 years	FFQ *	Sugar-sweetened beverages: Direct association
Mâsse et al., 2014^28^	n=11,385 12-19 years	Questionnaire*	Sugar-sweetened beverages: Direct association
Nasreddine et al., 2014^27^	n=868 6-19 years	24-hour record	Sugar-sweetened beverages: Direct association
Schröder et al., 2014^12^	n=1,149 10-18 years	24-hour record	Soft drink: Direct association
Vanderlee et al., 2014^31^	n=10,188 13-18 years	Questionnaire*	Sugar-sweetened beverages: No association

N: number of subjects; FFQ: Food Frequency Questionnaire; *Validated questionnaire; **Not mentioned in questionnaire validation.

**Table 2:** Cross-sectional studies (n=7) that analyzed the association of milk and sugar-sweetened beverages intake with body mass index in adolescents.

Reference	Sample size and age groups	Food survey and validations	Association between beverage intake and body mass index
Abreu et al., 2014^43^	n=1,209 15-18 years	FFQ*	Milk: No association
Gates et al., 2013^11^	n=443 9-18 years	24-hour record and questionnaire*	Milk: Inverse association
Liu et al., 2012^44^	n=14,332 2-19 years	24-hour record	Milk: Direct association Sugar-sweetened beverages: Direct association
Fayet et al., 2013^45^	n=4,487 2-16 years	24-hour record	Milk: No association
Albar et al., 2014^13^	n=636 11-18 years	Food diary	Milk: Inverse association Soft drink: Direct association
Beck et al., 2014^46^	n=319 8-10 years	FFQ*	Milk: Inverse association Soft drink: Direct association
Nassar et al., 2014^47^	n=190 16-18 years	Questionnaire*	Milk: No association Sugar-sweetened beverages: No association

N: number of subjects; FFQ: Food Frequency Questionnaire; *Validated questionnaire.

**Table 3:** Randomized clinical trials, cohort, case-control, quasi-experimental studies (n=8) that evaluated the association of beverages intake with body mass index in adolescents.

References and study design	Sample size and age groups	Food survey and validations	Association between beverage intake and body mass index
Stoof et al., 2013^48^ Coorte	n=238 13 years	24-hour record	Sugar-sweetened beverages: No association
Ebbeling et al., 2012^49^ Ensaio clínico aleatório	n=224 14-16 years	FFQ*	Sugar-sweetened beverages: No association
Laska et al., 2012^32^ Coorte	n=723 11-17 years	24-hour record	Diet soft drink: Direct association
Rhee et al., 2012^50^ Caso-controle	n=2,045 18-86 years	FFQ*	Sugar-sweetened beverages: Direct association
Ambrosini et al., 2013^51^ Coorte	n= 433 14-17 years	FFQ*	Sugar-sweetened beverages: Direct association
Jensen et al., 2013^29^ Quase experimental.	n=1,465 4-18 years	Questionnaire*	Sugar-sweetened beverages: Direct association
Jensen et al., 2013^52^ Coorte	n=324 6.9-13.0 years	Food diary	Sugar-sweetened beverages: No association
Vericker, 2014^53^ Coorte	n=1,550 13-14 years	Questionnaire*	Sugar-sweetened beverages: No association

N: number of subjects; FFQ: Food Frequency Questionnaire; *Validated questionnaire.

In order to evaluate possible risks of bias between studies, the instruments used to measure the intake of beverages was analyzed, being it a 24-hour record, the food frequency questionnaire (QFA) or any other tool. In the case of the questionnaire, its validation was checked and assured. Also regarding instruments and anthropometry, possible self-registration of data was taken into account, or data collection by trained researchers. In order to evaluate other possible bias risks in each study, the item limitation, to cover the limitations of each article chosen for the literature review, was included in the data collection tool.

## RESULTS

**Figure 1:**
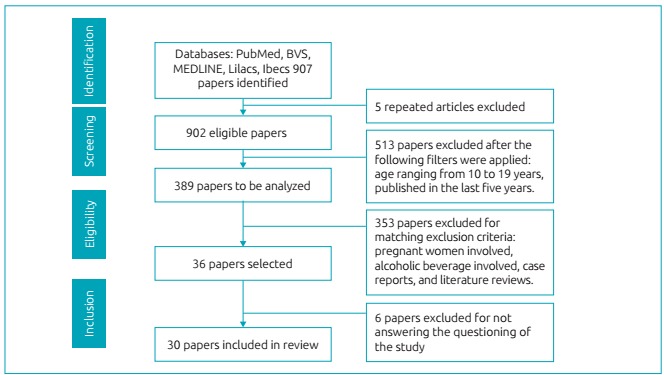
Research flowchart: identification, screening, eligibility, and inclusion of scientific papers in systematic review, according to PRISMA (2009).

The search with descriptors, according to the mentioned combinations, first retrieved 907 articles. Five repeated articles were removed at screening. After filtering and limiting them, 513 papers were excluded, with 389 articles remaining eligible. After reading of titles and abstracts, the following exclusion criteria were applied to 353 articles: pregnant women, alcoholic beverages, case reports, and literature reviews. A total of 36 eligible papers were read, of which six were excluded for not answering the research questioning. Finally, 30 studies that answered the guiding question of the investigation were included in the sample (Figure 1).

The studies had samples that, together, included 77,869 subjects. The 30 papers selected presented international samples. No national articles were found addressing the topic in question and matching the predefined criteria. Among the selected, 22 articles had cross-sectional design and were characterized as level 4 of evidence, corresponding to 73.3% of the total. Five papers were cohort studies; one was a case-control; and another one was quasi-experimental: these were level 3 and accounted for 23.3% of the total. One paper was a randomized clinical trial classified as level 2 of evidence. The 30 relevant articles were divided into three tables. Table 1 lists 15 articles with cross-sectional design, of which 11 addressed sugar-sweetened beverages in general, such as soft drinks, various types of juice, teas, and sports drinks, while 4 of them investigated only soft drinks. Table 2 shows seven cross-sectional articles dealing with sugar-sweetened beverages or milk intake, and Table 3 lists eight studies with different designs: cohort, randomized clinical trial, case-control, and quasi-experimental.

Among the studies evaluated, 22 (73.3%) reported association of the beverages intake with increased BMI. In all 30 articles together, 34 types of beverage were evaluated: four articles addressed two types of beverage in the same study, i.e., milk and sugary drinks. In the analysis comprising 34 beverages, 19 of them (56%) were shown to have a direct association with increase in BMI; six of them reported an inverse association, with reduction of BMI; and, finally, eight studies (26.7%) on nine types of beverage showed no association between their intake and BMI.

Among 30 papers selected for review, 18 (60.0%) addressed the relationship between sugar-sweetened beverages in general and BMI; five (16.7%) addressed only soft drinks intake; three (10.0%) dealt only with milk intake; two (6.7%) of them were on the association of soft drink or milk with BMI; and two (6.7%) of them reported the relation between sugar-sweetened beverages in general or milk and BMI. Among all studies, 20 of them approached on sugary drinks; 7 exclusively addressed soft drinks and seven, milk.

From the 20 studies (66.7%) that addressed sugary drinks in general, 55% reported an association between their intake and increased BMI. The seven studies on soft drinks agreed 100% in their findings that there was an association between intake and increase in BMI, and one of them considered soft drinks in diet version in the analysis. Finally, from seven studies on milk, only one (14.3%) showed association between its intake and increased BMI, while three (42.8%) reported protection from milk consumption against increase in BMI, and three found no association with increased or decreased BMI.

Regarding level-4 evidence and cross-sectional studies, in which a positive association between consumption and BMI was found, eight (57.1%) were related to the intake of sugar-sweetened beverages in general; six (42.8%) were related only to soft drink intake; and one (7.1%) involved milk consumption. Among level-3 studies, in which an association between consumption and increased BMI was identified, 75% addressed the ingestion of sugary beverages in general. In 42.9% of level 3 studies, there was no association between sugary beverages and BMI. Only one paper in this review was a randomized clinical trial in which the intake of sugar-sweetened beverage was not associated with BMI.

## DISCUSSION

First to mention, we have seen a lack of national papers on the subject. It should be emphasized, though, that research on this topic is fundamental to discuss the association between BMI and the intake of these beverages.

Most studies were cross-sectional (73.3%), characterized as level 4 of evidence. Thus, the conduction of other types of study favoring the identification of risk factors for increased BMI in adolescents should be encouraged.^12^ Despite the smaller number of studies that did not report association between these beverages and increase in BMI, 50% of them were level 3 or 2 of evidence, one of which was a randomized clinical trial with a high level of evidence, and three were cohort studies (37.5%).^23^ Further studies on drinks and BMI are therefore needed in order for a stronger conclusion to be drawn. The systematic review by Malik et al. shows evidence that the non-intake of sugary beverages allows control and reduction of adiposity indicators.^25^ Another systematic review published in 2013 pointed out signs to the establishment of a positive association between sugar-sweetened beverages and increased BMI. In the same review, however, one of the three articles selected did not report a significant association between BMI and their consumption.^26^

Regarding the stratification of beverages, the sugar-sweetened group had the highest number of publications (66.7%). There is an obstacle to identifying the beverages analyzed, as the term “sugar-sweetened drink” involves a variety of products, making it difficult to sort and draw conclusions about each drink. Although there were more papers about sugary drinks, soft drinks, when analyzed in isolation, were mostly associated (100%) with increase in BMI, and this is a red flag for the drawbacks of their intake.

After evaluating the studies, we noticed that in order to measure the intake of beverages, general and food frequency questionnaires (FFQ) were frequently applied (60.0%), as well as food diaries (30%). Two articles (7.0%) used food diary and one applied an FFQ along with a 24-hour record, which strengthens knowledge about dietary habits. The negative points of these data collection instruments are worth noting: the 24-hour record is often underreported, as it depends on memory and there is the difficulty in estimating portion sizes, thus not representing the actual variability of day-to-day food intake.^11^ ^,^ ^27^ In some cases, questionnaires lacked information about their validation. Among 19 articles using questionnaires and FFQ, 84% had been validated. On the other hand, 24-hour reports and food diaries require no validation, only the annotation of all foods and beverages consumed by either the researcher or the participant.

The questionnaire being self-administered or administered by trained researchers was also something to considered. From the total of questionnaires applied, 26% were self-reported by adolescents. Self-resport can induce errors that tend to mask or attenuate existing associations.^28^ In addition, in two articles the questionnaire had been applied by phone call. It is known that questions answered by phone can have memory bias, sub-registration, and social desirability.^29^ ^,^ ^30^ In other cases, a record was applied in person. The data collection instrument is a quality control measure when administered by trained researchers.^27^ In the study by Albar et al., the food diary was kept for more than four consecutive days, during which a trained researcher visited each participant three times at their residence. This type of investigation allows greater food variability. The trained researcher’s visit makes it possible to review the diary, deal with problems, edit possible flaws - such as omissions -, and include more details.^13^

Regarding information collection to obtain BMI, there were also variations. In some studies, weight and height were obtained by self-report,^28^ ^,^ ^31^ which leads us to the complexity in obtaining consistent conclusions, considering the tendency to overestimate height and underestimate weight.^11^ In addition, the range of instruments used for data collection is an obstacle to the homogeneity of discussion about the findings.

As to sample representativeness and randomness, 19 (63.3%) studies had representative samples and 20 (66.7%) had random samples. Thus, it is difficult to judge whether the data collected in this group of articles were representative of a population.^32^ The representativeness and randomness of samples are fundamental from the statistical point of view in order to extrapolate the information collected to the population.^33^

The limitations of this study are related to the difficulty in drawing conclusions from articles that, for the most part, do not evaluate individual follow-ups, the variety of collection instruments or the way they are applied. In addition, some articles do not bring descriptions of the randomness and representativeness of their samples.

The potential of this work is the detailed analysis of papers selected as to the evaluation of: relationship between sugary beverage or milk consumption and BMI of adolescents, the instruments used in studies, the randomness and representativeness of their samples, the way surveys were carried out, and how anthropometric data were measured.

One can conclude that there is no consensus in the literature about the association between the intake of sugar-sweetened non-alcoholic drinks or milk and the BMI of adolescents. The overall rate of consumption of sugar-sweetened non-alcoholic beverage by adolescents is high, and additional follow-up studies should be conducted to elucidate these effects on adolescents’ BMI and overall health.
